# Revolutionizing heart disease prediction with quantum-enhanced machine learning

**DOI:** 10.1038/s41598-024-55991-w

**Published:** 2024-03-29

**Authors:** S. Venkatesh Babu, P. Ramya, Jeffin Gracewell

**Affiliations:** 1 Department of CSE, Christian College of Engineering and Technology, Dindigul, India; 2 Department of AI and DS, PSNA College of Engineering and Technology, Dindigul, India; 3 Department of Electronics and Communication Engineering, Saveetha Engineering College, Chennai, India

**Keywords:** Quantum computing, Machine learning, Ensemble methods, Heart disease prediction, Computational biology and bioinformatics, Machine learning

## Abstract

The recent developments in quantum technology have opened up new opportunities for machine learning algorithms to assist the healthcare industry in diagnosing complex health disorders, such as heart disease. In this work, we summarize the effectiveness of QuEML in heart disease prediction. To evaluate the performance of QuEML against traditional machine learning algorithms, the Kaggle heart disease dataset was used which contains 1190 samples out of which 53% of samples are labeled as positive samples and rest 47% samples are labeled as negative samples. The performance of QuEML was evaluated in terms of accuracy, precision, recall, specificity, F1 score, and training time against traditional machine learning algorithms. From the experimental results, it has been observed that proposed quantum approaches predicted around 50.03% of positive samples as positive and an average of 44.65% of negative samples are predicted as negative whereas traditional machine learning approaches could predict around 49.78% of positive samples as positive and 44.31% of negative samples as negative. Furthermore, the computational complexity of QuEML was measured which consumed average of 670 µs for its training whereas traditional machine learning algorithms could consume an average 862.5 µs for training. Hence, QuEL was found to be a promising approach in heart disease prediction with an accuracy rate of 0.6% higher and training time of 192.5 µs faster than that of traditional machine learning approaches.

## Introduction

In the modern world, heart disease is being emerged as the prominent cause of human death. Changes in food habits, lifestyle, and work-related stress are the major contributors to the increase in the rate of heart disease. The World Health Organization (WHO) stated that 17.7 million people all around the world have been suffered from heart disease every year^1^. According to the report released by Global Burden of Disease in 2016, 1.7 million Indians were affected by cardiovascular diseases (CVD)^2^. Coronary artery disease (CAD) is the most common type of CVD and the reason behind CAD is blockage or obstruction in at least one of the coronary arteries. Therefore, a lack of blood supply will damage the heart, brain, and legs which in turn causes heart attacks and rupturing of blood vessels. Approximately 2% of people around the world are suffering from CAD and 2% of the annual budget has been spent on CAD treatment^3^. The heart-related disease like CAD increases the spending on health care and reduce the productivity of the individual. Hence, there is a need for precise preventive and diagnostic mechanisms to reduce the mortality rate of CAD.

Normally, diagnosis of heart disease can be done by manual examination of the risk factors such as patient’s age, sex, family history, lifestyle, etc., physical examination reports, and analyzing the patient’s symptoms. But the manual examination leads to inaccurate prediction since some parameters to be analyzed may remain hidden and it is computationally expensive to analyze such huge factors^4^. Angiography is widely used as the most precise method for diagnosing CAD^5^, but, it is associated with high cost and major side effects. Healthcare sectors are struggling to offer reliable diagnoses at a reasonable cost. Moreover, image-based detections are more costly and not suitable for screening large populations. So, many researchers have endeavored to develop a non-invasive, economical, and fast automated diagnosis system for early detection of CAD based on Machine Learning Algorithms.

Many ML algorithms^5–11^ have shown promising results in terms of greater accuracy rate and speed up the performance in early diagnosis of heart disease by identifying hidden patterns. Despite these greater benefits, still there is a computational bottleneck when dealing with larger and more complex data using traditional computers results ML algorithms being incapable of handling computationally rich tasks^12^. At this point, the quantum computing principle has lent its hand to enhance computational power. Thus, quantum Enhanced machine learning algorithms facilitate healthcare industries to evaluate and treat complicated health disorders.

The major contribution of this article is conducting an experimental study about various ML algorithms that have been utilized recently in predicting heart disease from which suitable ML methods have been identified. Then this study revealed the essentials of quantum computing integration to enhance the machine learning algorithms to speed up the computation process. Then, the proposed QuEML techniques have been evaluated based on selected features that show greater accuracy and speed in prediction.

The paper begins by providing an overview of the various machine-learning algorithms used for heart diagnosis which is given in “Introduction”. This is followed by a detailed study of ML and QML algorithms to explore their strengths and weakness in detecting disease are explained in “Background of study”. Further, an explanation of the workings of Quantum Enhanced Machine Learning Algorithms (QML) is presented in “System materials and methods”. The experimental results, obtained from utilizing the datasets, are then presented in “Results and discussions” to demonstrate the effectiveness of the diagnostic methods. The paper concludes by summarizing the findings and highlighting potential areas for future improvement which is presented in “Conclusion”.

## Background of study

This section portrays the various ML techniques that have been employed by various academicians for effective heart disease diagnosis. The major reason to utilize the ML algorithm is that it is capable of detecting hidden patterns and can operate with large datasets to make predictions.

In^13^, Syed et al. were involved in developing SVM-based Heart Disease Diagnosis using the datasets Cleveland, Hungarian, Switzerland, and a combination of all of them (709 instances). Then, utilize the advantages of Mean Fisher Based Future Selection and Accuracy-based Feature selection algorithms for optimal feature selection. Further, the selected feature subset is refined through Principal Component Analysis. Finally, Radial Basis Function Kernel Based Support Vector Machine is applied over the reduced feature subset to categorize the heart disease patient from normal people. Their experimental result demonstrated that the proposed framework outperforms with an average accuracy rate of 85.3%. Youn-Jung et al.^14^ have chosen the SVM algorithm as it can handle high dimensionality problems to detect heart patients where data about patients are collected from University Hospital through a self-reported questionnaire and the experiment is carried out based on leave-one-out cross-validation (LOOCV) and it was proven that SVM based classification is a promising approach with the highest detection accuracy of 77.63%. Ebenezer et al.^15^ have developed a mechanism based on Boosting SVM to enhance prediction accuracy by combining the results of all weak learners. For reducing misclassification, normalization, redundancy removal, and heat-map are applied over the given datasets. Upon applying heat-map, it identifies important factors such as age and maximum heart rates in predicting heart disease which further facilitates prediction. In this study, Cleveland datasets were used which contain 303 instances with 13 attributes. Through the experimental results, the performance of the Boosting SVM is compared with Logistic regression, Nave Bayes, decision trees, Multilayer Perceptron, and random forest. Out of which, the proposed Boosting SVM achieves greater accuracy of 99.75%.

Medhekar et al.^16^ were involved in developing a Naïve Bayes Classifier (NBC) based Heart Disease Prediction System using the Cleveland dataset downloaded from the UCI repository to classify the patients into five categories viz. no, low, average, high, and very high to identify the severity level of disease. Then, System accuracy was calculated and the results are tabulated to evaluate the system performance through which it can be observed that the proposed NBC-based system could attain 88.96% of accuracy. Vembandasamy et al.^18^, proposed a framework to detect heart disease using NBC. The experiment is carried out by the WEKA tool over the datasets collected from a diabetic research institute in Chennai and the accuracy rate yielded by the system is about 86.4198%. The authors of^17^ presented an article for heart disease detection using NBC and could able to attain an accuracy rate of 80% which is comparably poor by performing prediction over the dataset collected from Mayapada Hospital which contains 60,589 records. Heart disease prediction using NBC is quite challenging since all the properties in NBC are required to be mutually independent^15^.

The authors of^19–21^ have employed the concept of neural networks in heart disease diagnosis to improve the accuracy further. In^20^, Firstly, Cleveland datasets were subjected to a Feature selection algorithm that uses information gain to remove the features which do not contribute to the disease prediction. Further, the ANN algorithm was applied over the reduced feature set for classification. This study dictated that the accuracy (89.56%) of the system with a reduced feature set (8 features) is slightly improved than the accuracy (88.46%) of the system with a full feature set (13 features). Miray et al.^19^ have presented an intelligent heart disease diagnosis method using a hybrid Artificial Neural Network (ANN) and Genetic Algorithm (GA) where GA is used to optimize the parameters of ANN. Experimental results are obtained by using Cleveland data through which it is visible that the hybrid approach outperforms Naive Bayes, K- Nearest Neighbor, and C4.5 algorithms in terms of accuracy rate (95.82%), precision (98.11%), recall (94.55%) and F-measure (96.30%). Even NN model is good at generalizing data and capable of analyzing complex data to discover hidden patterns, many medical experts are dissatisfied with NN because of its black-box characteristics. That is, NN models get trained without knowing the relationship between input features and outputs. So, if many irrelevant features are used to train the NN model, it results in inaccurate prediction in testing. To address this challenge, Kim and Kang^21^ have employed two preprocessing steps before applying ANN. The first step is the feature selection step to select the features based on ranking. Then, feature correlation analysis is performed to make the system learns about the correlation between feature relations and NN output thereby eliminating the black-box nature. The overall experiment is performed on the Korean dataset containing 4146 records and resulted in a larger ROC curve with more accurate predictions. However, ANN could be suffered from data overfitting and temporal complexity and it may fail to converge when dimensionality is low.

As K-Nearest Neighbor (KNN) is a simple and straightforward approach where samples are classified based on the class of nearest neighbors, the authors of^22^ have employed the KNN algorithm for classifying heart disease. Since medical datasets are larger, the Genetic algorithm was utilized to prune redundant and irrelevant features from 6 different medical datasets taken from the UCI repository to improve the prediction accuracy which is 6% greater than the accuracy rate achieved by the KNN algorithm without GA. Ketut et al.^23^ have proved that simple and fewer features are good enough to reduce misclassification, especially in heart disease prediction. In the experimental study, chi-square evaluation is done over the given Hungarian data set which contains 293 records with 76 parameters. But, in this paper, only 13 parameters were taken into consideration and after performing a chi-square evaluation, it results in 8 parameters as the most important parameters. Subsequently, KNN is executed with reduced feature set results with 81.85% of accuracy which is considerably greater than NBC, CART, and KNN with full feature set.

A heart disease prediction model using Decision Tree Algorithm (DT) has been implemented on UCI datasets^24^. The main aim of this paper is to reveal the importance of the pruning approach in DT which provides compact decision rules and accurate classification. The J48 DT algorithm is implemented with three types such as DT with pruning, un-pruning, and pruning with a reduced error rate. In this experiment, it shows fast blooding sugar is the most important attribute which yields greater accuracy (75.73%) than other attributes but, it is comparably very poor. The DT algorithm is simple, but it is capable of handling only categorical data and it is inappropriate for smaller datasets and datasets with missing values^25^.

In Research^26^, Logistic Regression (LR) is applied to UCI datasets to classify cardiac disease. Initially, data preprocessing is done to filter the missing values, and a feature selection process based on correlation is carried out to select the highly co-related features. Then given data is split into training and testing splits to perform classification by LR. From the tabulated results, it shows that LR increases the accuracy by 87.10% when increases the training size from 50 to 90%. Paria and Arezoo^27^ were involved in developing a regression-based heart attack prediction system. For this purpose, three regression models were made based on the variable selection algorithm and applied to the dataset with 28 features collected from Iran hospitals. The model that uses the following features such as severe chest pain, back pain, cold sweats, shortness of breath, nausea, and vomiting yielded a greater accuracy of 94.9% than that of the model using physical examination data and ECG data.

Yeshvendra et al.^28^ have employed Random Forest (RF) algorithm for heart disease prediction. In this paper, the Cleveland heart disease dataset was exploited which has non-linear dependency attributes. So, RF is considered the optimum solution for the non-linear dependent datasets and it produced good accuracy of 85.81% by making a bit of adjustment over the non-linear dataset. To reduce the overfitting problem, Javeed et al.^29^ have developed an Intelligent Heart Disease Diagnostic system that uses Random Search Algorithm (RSA) for feature selection from the Cleveland dataset and Random Forest (RF) model for heart disease prediction. Based on the experimental results, it is observed that RSA-based RF produced 93.33% accuracy using only 7 features which are 3.3% higher than the conventional RF. As the ensemble nature of RF is capable of producing high accuracy, handling missing and huge data, eliminating the need for tree pruning, and solving the problem of overfitting, authors of^30^ have employed RF to predict heart disease. In addition, chi-square and genetic algorithms were applied to select the prominent features from the heart disease data set collected from various corporate hospitals in Hyderabad. The performance of the proposed system is about 83.70% of accuracy which is considerably greater than the NBC, DT, and Neural Nets.

Jafar and Babak^31^ proposed an efficient and accurate system to diagnose heart disease. This research developed an ensemble classification model based on a feature selection approach. The heart disease dataset used by this research is downloaded from the UCI repository which contains 270 records with 13 useful variables. After selecting the prominent features, seven classifiers namely SVM, NBC, DT MLP, KNN, RF, and LR were used in ensemble learning to predict the disease. The final prediction over the given sample is done by combining the prediction result of all seven classifiers using the Stacked Ensemble method. An ensemble learning based on a genetic algorithm had shown the best performance and could lead to 97.57% accuracy, 96% sensitivity, and 97% specificity. Ensemble learning is a combination of multiple classifiers which improves the predictive performance by combining the output of individual classifiers. To identify the best ensemble method in heart disease detection from the heart Stalog dataset, Indu et al.^32^ developed an automatic disease diagnosis system based on three ensemble learners such as Random Forest, Boosting, and Bagging along with PSO-based feature subset selection method. The overall experiment is carried out using RStudio and the proposed system with the bagging approach yielded greater accuracy than the other approaches. The Table 1 summaries the major finding.

**Table 1:** Major findings of the study.

S. no.	References	Algorithms	Major findings
1	^13–15^	SVM	SVM is most preferred as it can handle high dimensionality problems Boosting SVM enhances accuracy up to 99.75%
2	^16,17^ ^18^	NBC	NBC's heart disease prediction is challenging. Since requires all features to be mutually independent
3	^19,20^ ^21^	ANN	ANN is good at generalization and capable of analyzing complex data and able to attain an accuracy of up to 95.82% To eliminate the block box nature of ANN, feature selection is employed which enhances prediction accuracy further
4	^22,23^	KNN	KNN is simple and not suitable for high dimensional data which is common in heart disease datasets In high-dimensional data, the distance between points tends to become less informative, making it difficult for KNN to identify the nearest neighbors accurately
5	^24,25^	DT	It can be prone to overfitting and it is inappropriate for handling data with missing values DT can sometimes be biased toward selecting features resulting in a less accurate model
6	^26,27^	LR	It requires a linear relationship between features It can limit the capability to capture underlying patterns leading to lower accuracy
7	^28,29^ ^30^	RF	The Potential issue with RF is that it can be computationally expensive and memory-intensive, particularly when dealing with medical data
8	^31,32^	Ensemble Approaches	The Bagging Ensemble approach yielded greater accuracy up to 97.57% due to its ability to reduce overfitting, improve generalization, and combine the strengths of multiple machine learning algorithms
9	^36–40^	Quantum Computing	It performs certain operations faster This speedup can be harnessed to accelerate certain machine-learning algorithms by reducing the time required for training

### Major findings of the study

- Automatic heart diagnostic systems that were developed using ML techniques were surveyed as heart disease is the major cause of human deaths in today’s world, so an effective and accurate diagnosis system is to be developed to save human lives.
- From the above study, it is observed that many researchers were interested in machine learning for heart disease diagnosis since it helps to reduce diagnosis time and increases accuracy.
- From the study, every new approach competes with one another to win a greater accuracy rate.
- Boosting-based SVM and an Ensemble of classifiers are being seen as the most promising methods that yielded the greater accuracy ever seen.
- One algorithm may work well for one dataset while cannot work well for another dataset.
- The accuracy of the system may rely on the quality of the datasets used.
- Some datasets can have missing values, redundancies, and noises which makes data to be unsuitable. Such uncertainty can be resolved by applying data preprocessing techniques such as normalization, missing value imputation, etc.
- Some datasets may have too many attributes which may threaten the performance of ML in accuracy and computational time. It can be improved by applying suitable feature selection strategies to perform prediction with the most informative features.

As machine learning algorithms predict the output by learning the relationship between input features and class labels based on classical theories of probability and logic, the accuracy rate is still on the lower side^33,34^. So, it requires a lot of improvements to have general acceptability for disease prediction. Another major issue in traditional machine learning algorithms is computation time. Since the computation time is increased with an increase in the size of the feature set. Therefore, the main aim of the paper is to enrich the performance of classical ML algorithms and make them outperform all the baselines in terms of Precision, Recall, F-Measures, and Computation Time^35^. It redirects the research toward quantum computing to create a pave to integrate quantum computing with ML approaches.

### Motivation for the study

After having detailed observation from the recent articles^36–40^, quantum mechanics have shown excellent performance in various fields such as classification, disease prediction, object detection, and tracking and achieved remarkable performance over classical probability theory-based models. The basics of quantum computing, its essential features, and working are available in public domains, as a result, it cannot be explored further.

When compared with traditional machine learning algorithms, quantum Enhanced machine learning algorithms are capable of reducing training time, automatically adjusting the network hyper-parameters, performing complex matrix and tensor manipulation at high speeds, and use of quantum tunneling to achieve objective function goals. Integrating quantum computing and machine learning enables healthcare sectors to evaluate and treat complicated health disorders. Quantum computing uses the principle of quantum physics in which a single bit can be represented in both 0 and 1 which is known as qubits (Quantum Bits). Another salient feature of quantum computing is Superposition allows the particle to exist in multiple states at a time which provides tremendous power to handle massive amount of data, Entanglement occurs when pair of particles are generated which allows them to share spatial proximity or interact, Quantum tunneling enables computer to complete the task faster and Quantum gates work on collections of quantum sates to produce desired output. The first quantum computing device came into act in the year of 2000, so many researchers recently utilized quantum computing principle to analyze billions of diagnostic data with the help of artificial intelligence techniques. Quantum-enhanced machine learning assists physicians with earlier and more accurate health disease predictions. According to the report of^41^, the time spent on research and analyzing diagnostic data will decrease when quantum computing is integrated with healthcare systems.

With this motivation, the paper aims to implement Quantum Enhanced Machine Learning approaches for diagnosing heart diseases and it yields a remarkable accuracy rate and computation time shown in "Results and discussions" by simply replacing classical probability theory with quantum probability theory and it makes use of superposition state that provides a higher degree of freedom in decision making.

## System materials and methods

### Dataset description

It describes the datasets that have been used to implement QuEML (Quantum Enhanced Machine Learning) Framework for heart disease diagnosis. The Heart Disease Dataset (HDD) is taken from the Kaggle repository and it is a comprehensive dataset of 1190 instances which is a combination of five different datasets listed in Table 2 and the combination is based on 11 common features with one target variable listed in Table 3.

**Table 2:** Datasets.

S. no.	Dataset	Instances
1	Cleveland	303
2	Hungarian	294
3	Switzerland	123
4	Long beach VA	200
5	Stalog heart dataset	270

**Table 3:** Description of dataset features.

Type	Name	Description	Range
Demographic	Age	Patient’s age in years	28 to 77
Demographic	Sex	Patient’s gender; 1-male; 0-female	0 or 1
Symptom and examination	Chest pain type	1-typical; 2-typical angina; 3-non anginal pain; 4-asymtomatic	1 to 4
Symptom and examination	Resting blood pressure	Level of blood pressure at resting mode in mm/Hg	0 to 200
Laboratory and echo	Cholesterol	Serum cholesterol in mg/dl	0 to 603
Laboratory and echo	Fasting blood sugar	Blood sugar level on fasting > 120 mg/dl; 0-no; 1-yes	0 to 1
ECG	Resting ECG	Result of ECG at rest; 0-normal; 1-ST-T wave abnormality; 2-showing probable or definite left ventricular hypertrophy by Estes’ criteria	0 to 2
ECG	Maximum heart rate	Maximum heart rate achieved	60 to 202
Symptom and examination	Exercise angina	Angina induced by exercise; 0-no; 1-yes	0 to 1
ECG	Old peak	Exercise-induced ST depression in comparison with the state of rest	-2.6 to 6.2
ECG	ST slope	The slope of the peak exercise ST segment; 1-up sloping; 2-flat; 3-down sloping	0 to 3
Target variable		0-normal; 1-HD (heart disease)	0 or 1

### Proposed methodologies

**Figure 1:**
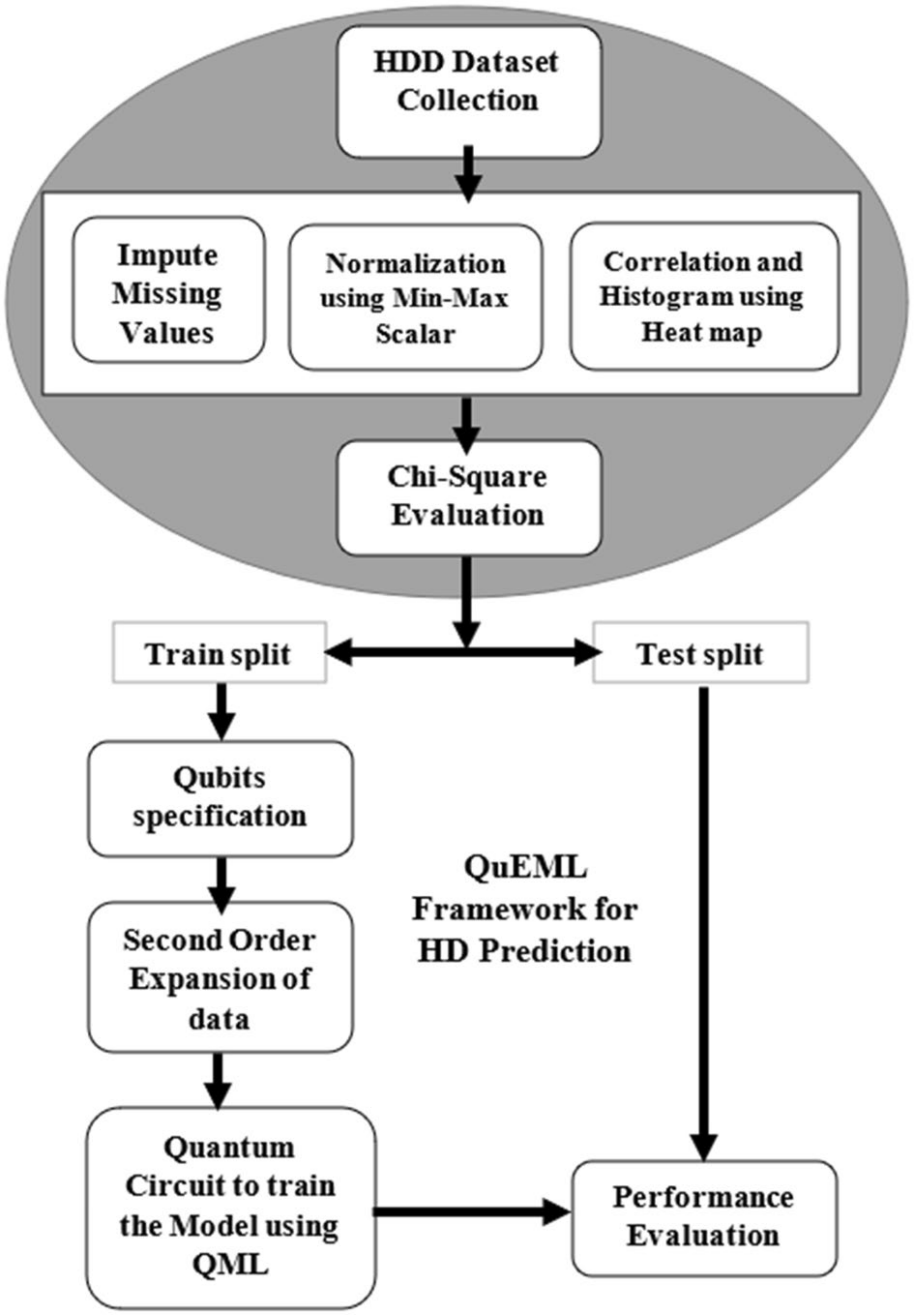
Steps used in QuEML.

The steps that are followed in this research are depicted in Fig. 1. The collected HDD is subjected to Exploratory Data Analysis (EDA) to eliminate outliers, impute missing values and normalize the dataset. Then preprocessed dataset is fed into Chi-square Evaluation to retain the top-ranked features (TRF). Then the data sets with TRF are split into training, and testing split with the ratio of 70:30. Now, the quantum-enhanced approach is developed based on training data in which the number of qubits is specified based on ${{\text{log}}}_{2}(No.of features)$. Further feature mapping can be carried out by entangling qubits to second-order expansion. At last, the quantum circuit is generated to train the model using QML approaches such as QSVM, QANN, and QBE. Thereafter, the performance of Quantum enhanced machine learning framework is evaluated against traditional machine learning approaches by test set based on several performance metrics such as Accuracy, Precision, Recall, and F1-Score. The overall process of proposed QuEML framework is depicted in Algorithm 1.

**Algorithm 1:**
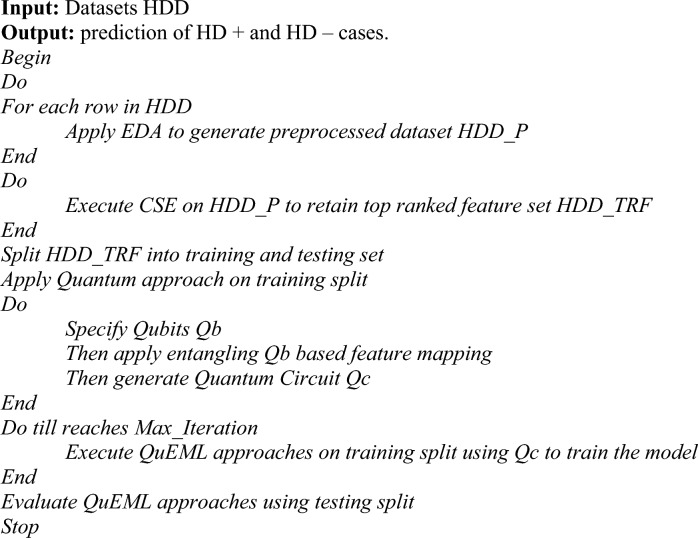
QuEML framework.

#### Exploratory data analysis (EDA)

**Figure 2:**
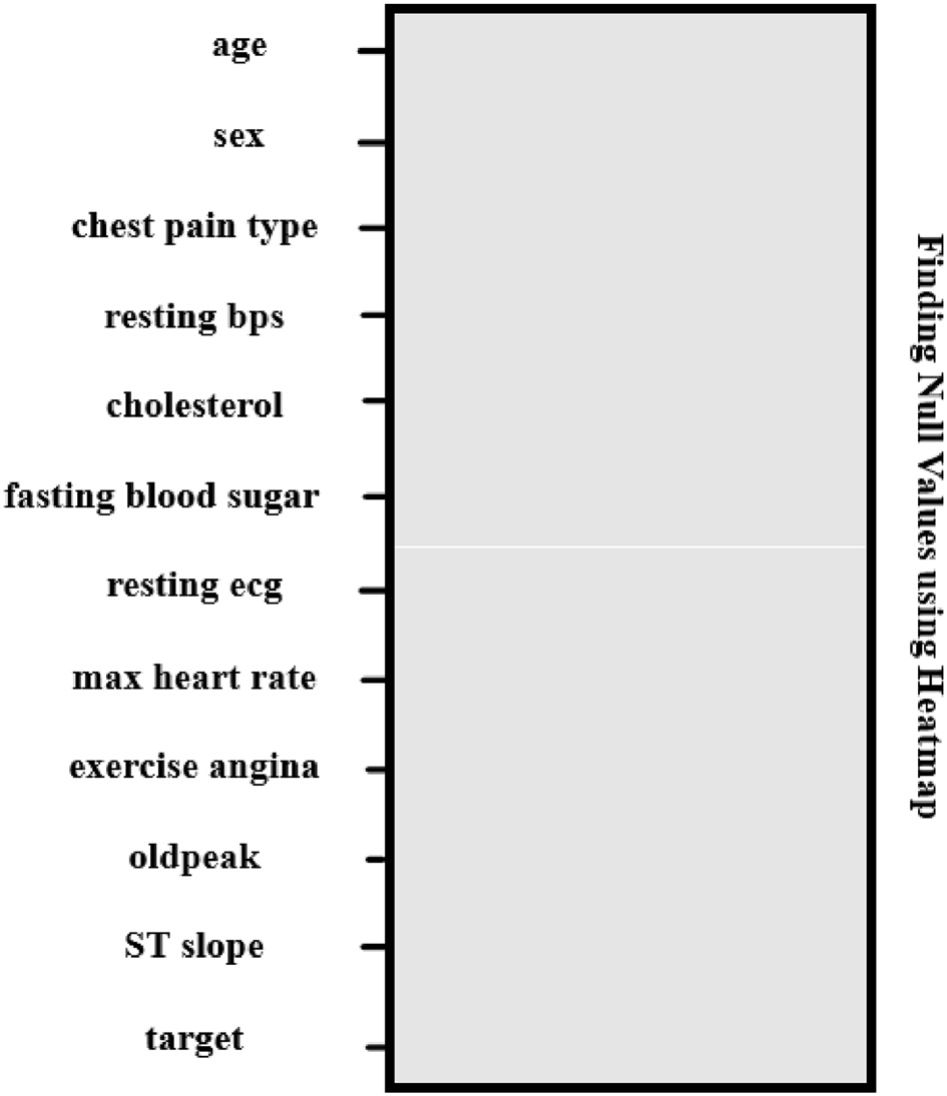
Finding null values.

**Figure 3:**
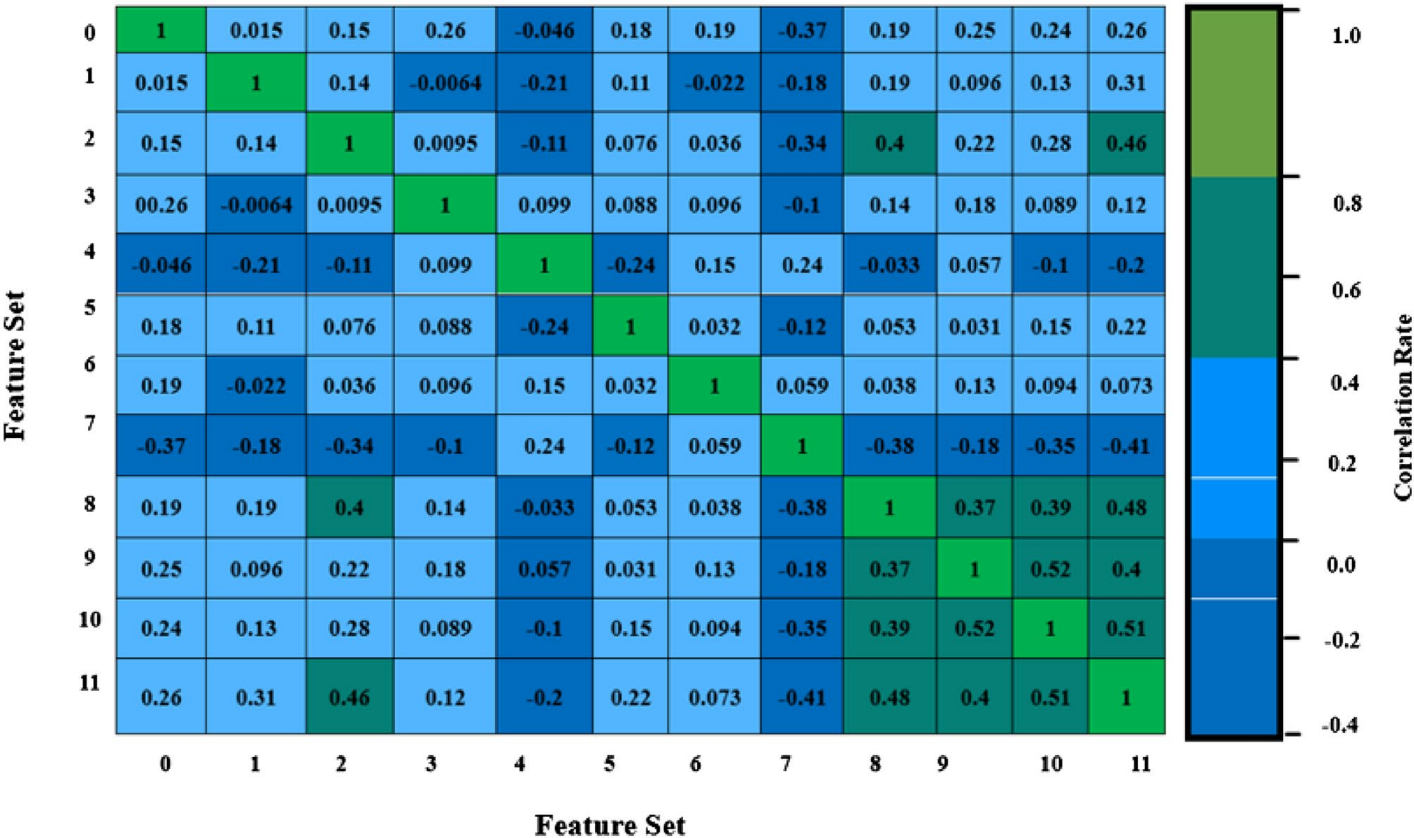
Features distribution.

It is considered an essential step in the data-driven analysis. It deals with the visualization of data with various aspects such as imputing missing values, normalization, and identifying highly correlated and low variance attributes. It can be done with the help of pandas and matplot libraries and the result is shown in Fig. 2 from which it has been inferred that the chosen dataset has no duplicate and null values and it is good which will be further analyzed. Subsequently, Min–Max scalar technique is applied over the given data set which rescales the data from its original range to a defined range [0, 1] using Eq. (1) where X’ is a rescaled value and X is an original value and various notations used in the following subsequent sections are given in Table 4. It can be done with the help of MinMaxScaler () from the sklearn library which further enables Quantum enhanced machine learning algorithms to treat the dataset equally. Finally, the important features can be inferred from the given data set by heatmap () which identifies the correlation between each feature as shown in Fig. 3.

**Table 4:** Notations with description.

S.No	Notation	Description
1	min(X)	Find minimum value in given data set X
2	max(X)	Find maximum value in given data set X
3	CSS	Chi square score
4	OF(i)	Observed frequency of feature ‘I’
5	EF(i)	Expected frequency of feature ‘I’
6	r_y_(ɵ)	Qubit rotation around y axis through an angle ɵ
7	< 0|σ_z_|0 > and < 1|σ_z_|1 >	States represented in Pauli-Z(σz) measurement
8	|ϕ(x′) >	Quantum states
9	v(ϕ(x′))	Quantum feature map
10	h^n^	Hadamard gate
11	C(r)	Control register
12	T(r)	Training register
13	Te(r)	Temporary register
14	Tt(r)	Test register
15	Ta(r)	Target register
16	S0	State of quantum registers
17	fi(x′)	Estimate of target variable
18	u(x,y) and u(x′)	Encode input data x and y into quantum state
19	< M >	Expectation measurement
20	N(T +)	Number of true positives
21	N(T−)	Number of true negatives
22	N(F +)	Number of false positives
23	N(F−)	Number of false negatives
24	P	Precision
25	A	Accuracy
26	R	Recall
27	Se	Sensitivity
28	Sp	Specificity
29	F	F1 score
30	T	Training time

1 $$\mathrm{X^{\prime}}=\frac{X-{\text{min}}(X)}{{\text{max}}\left(X\right)-{\text{min}}(X)}$$

In Fig. 3, the features which are listed in Table 3 are numbered from 0 to 11. The figure shows that the feature Chest Pain Type has a positive correlation with the target variable which indicates that people who have chest pain results in a greater chance to have severe heart disease. In addition to this, cholesterol and heart rate features have a negative correlation with the target variable.

#### Chi-square evaluation (CSE)

It picks certain features from the non-negative data set that has the best relationship with the target variable. After performing CSE, top K-features are chosen based on their chi-square score results from a reduced feature set as a large number of irrelevant features increase the training time exponentially and increase the risk of overfitting. In this research, the K value is set as 8 and the implementation of CSE is carried out by SelectKBest() of the sklearn library. The Chi-Square Score (CSS) is calculated between each feature and target variable by using Eq. (2) where OF(i) represents the Observed Frequency of feature ‘i’ concerning the target variable and EF(i) denotes the Expected Frequency of feature ‘i’ concerning target variable.

2 $${\text{CSS}}=\frac{{(OF\left(i\right)-EF\left(i\right))}^{2}}{EF(i)}$$

#### QuEML framework

Quantum Enhanced Machine Learning algorithms are employed in this research since it is optimizing traditional machine learning algorithms and helps in superior classification and pattern detection which in turn discover abnormal behaviors and eliminating fraudulent medical claims. From the study that has been conducted in "Background of study", it has been visualized that the traditional approaches such as SVM, ANN, and Ensemble approach had shown excellent performance in automatic heart disease diagnosis systems with greater accuracy rate. Hence, the proposed framework has utilized those traditional classifiers which are further optimized by applying quantum computing principles.

##### Quantum artificial neural network (QANN)

**Figure 4:**
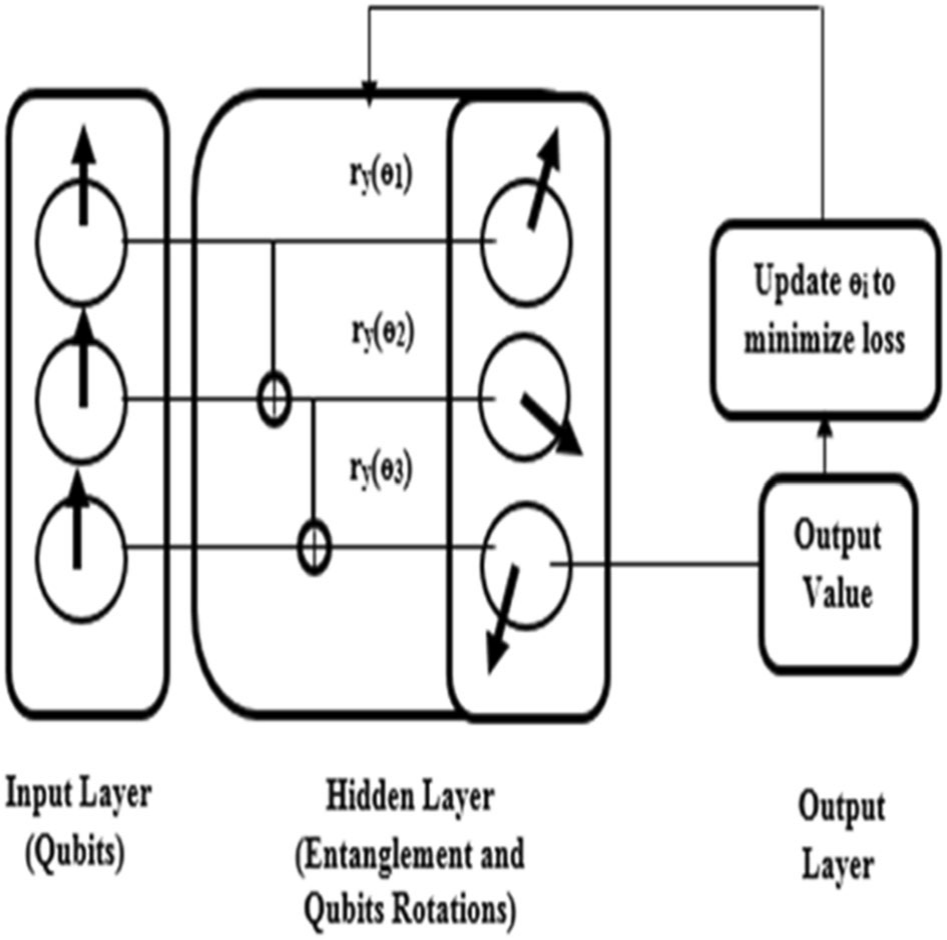
QANN architecture.

Training an ANN is a complex task since the parameters of the network must be optimized to avoid overfitting. When the number of parameters to be optimized is increased, it leads to a lot of computational overhead. So, such complex tasks can be easily handled by QANN. In QANN, the neurons of input layers are replaced by Qubits as shown in Fig. 4 which avoids the usage of nodes in hidden layers as in traditional ANN. Thus, it reduces the number of parameters to be optimized. In the proposed approach^42^, no of parameters to be optimized are 18 as there are six hidden layers with three single qubit rotation in each layer. The input data is encoded as the superposition of ${2}^{k}$ states where k denotes the dimension of qubits using an amplitude embedding scheme where input data is embedded as the amplitude of different states in superposition and the amplitudes are represented by a state vector with a dimension of 8 × 1. For example, in this research, no of Qubits are 3 (${{\text{log}}}_{2}\left(8\right)=3)$ and input data is encoded as a quantum state with a superposition of different states such as x_1_ |000 >  + x_2_ |000 >  + …. x_8_ |000 > . The hidden layer contains a connection between qubits that uses Controlled NOT (CNOT) gates r_y_(ɵ) for entanglement. r_y_(ɵ) is a single qubit rotation around the y-axis through an angle of ɵ. Quantum gates are used to modify the state of the qubits using rotational parameters. Finally, the output value is determined based on measuring the state of the qubit. The output value is measured by using Pauli-Z(σ_z_) measurement in which states are represented as < 0|σ_z_|0 > and < 1|σ_z_|1 > to determine the binary output 1 and −1 respectively. Here quantum measurement act as an activation function that saves computational effort. Then, the output value is compared with the target variable to calculate the loss. Then, rotational parameters are modified continuously until the state of qubits gives the desired target value.

##### Quantum support vector machine (QSVM)

**Figure 5:**
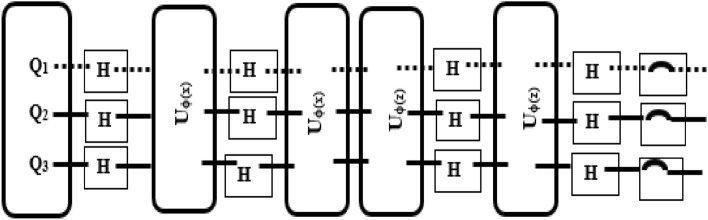
Estimating state overlap: depth = 2.

Quantum SVM offers a computational advantage over traditional SVM which further facilitates classification with higher dimensions. It can be trained as similar to traditional SVM once the quantum kernel is obtained. In QSVM^43^, the input data is transformed into quantum states |ϕ(x′) > . This can be achieved by performing quantum feature map v(ϕ(x’)) in which ϕ() is a classifier that can be applied to input data x’. Feature maps are done based on the Ansatz principle as given in Eq. (3) in which ‘h^n^’ denotes the hadamard gate which is applied to each qubit and ‘n’ indicates several qubits. Moreover, the depth of the circuit can be defined as ‘2’ which applies the Ansatz principle two times. Hence, the form of the feature map can be as shown in Eq. (4). Then, the Quantum kernel can be measured by estimating the overlap between two states |< ϕ(x′)|ϕ(z′) >|^2^ which can be done by using the circuit shown in Fig. 5 and the frequency of the measurement string like [0,0,0….] gives the estimation of state overlap. The parameterized quantum circuit is generated using Qiskit Aqua Library which uses the SecondOrderExpansion function that takes feature dimensions and depth as arguments. At last, Qiskit provides a predefined function through which training is performed by applying a quantum kernel with less computational cost, and the hyperparameters of QSVM are tuned to minimize the loss.

3 $${\text{v}}\left( {\phi \left( {{\text{x}}^\prime } \right)} \right) = {\text{u}}\left( {\phi \left( {{\text{x}}^\prime } \right)} \right) \otimes {\text{h}}^{{\text{n}}}$$

4 $${\text{v}}\left( {\phi \left( {{\text{x}}^\prime } \right)} \right) = {\text{ u}}\left( {\phi \left( {{\text{x}}^\prime } \right)} \right) \otimes {\text{h}}^{{\text{n}}} \otimes {\text{u}}\left( {\phi \left( {{\text{x}}^\prime } \right)} \right) \otimes {\text{h}}^{{\text{n}}}$$

##### Quantum bagging ensemble (QBE)

**Figure 6:**
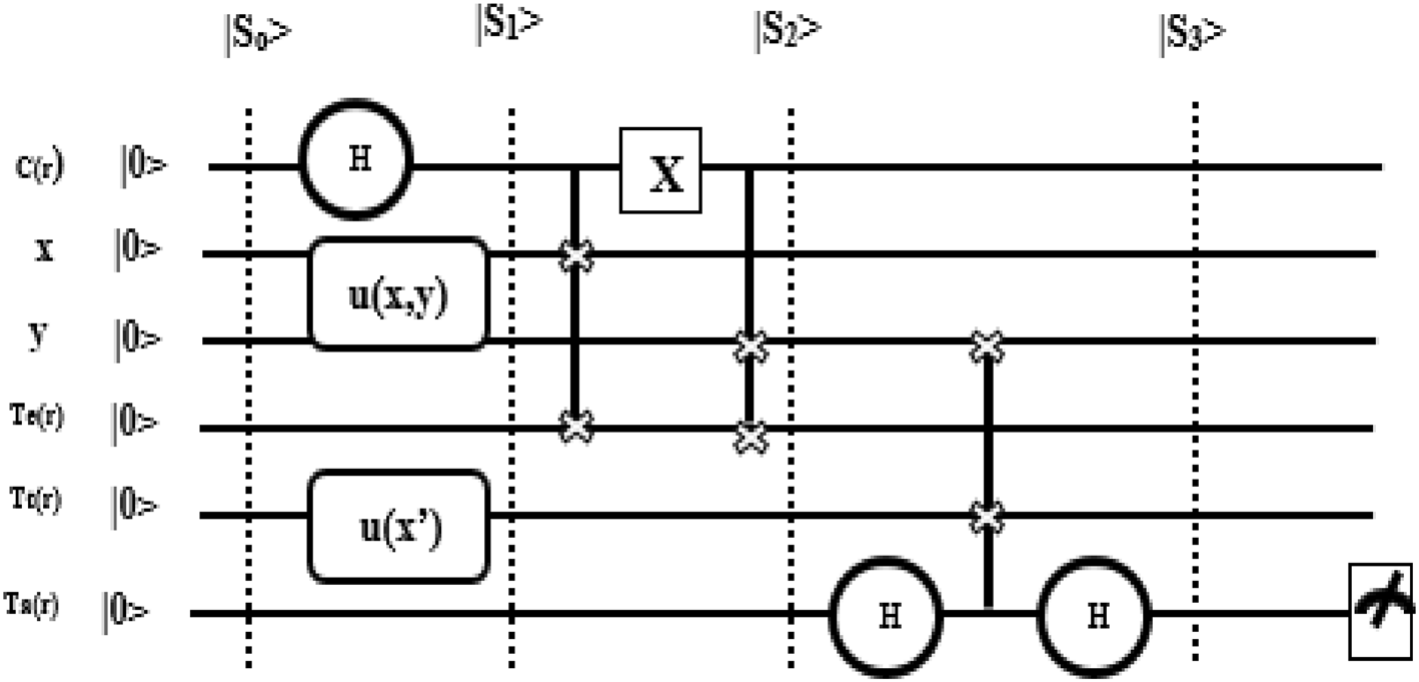
Quantum circuit for QBE.

It produces a combined model that outperforms a single model by combining the strengths of collections of simple weak learners which reduces the prediction error. In order to implement QBE^44^, 5 quantum registers (S_0_) are used such as Control register (C(r)), Training register (T(r)), Temp register (Te(r)), Test register (Tt(r)) and Target register (Ta(r)) as shown in Fig. 6 where the size of T(r) and Tt(r) depends on the size of the input, Te(r) determines the size of subsamples used as input in the single weak classifier, size of Ta(r) depends on the nature of target variable and size of C(r) determines the number of weak learners used in the prediction. Then, the proposed QBE begins with the state preparation process (|S_1_ >) where training and testing data are encoded into respective registers, and the control register is initialized into uniform superposition as given in Eq. (5) in which ‘h’ indicates Hadamard gate, ‘n’ indicates a number of qubits, u(x,y) and u(x′) are the unitary which encode the input data ‘x’ and ‘y’ into quantum state and S_0_ is the state of quantum registers. Further, sampling in superposition (|S_2_ >) is performed using Eq. (6) where quantum oracle ‘v’ is used which entangles several sub-samples of data (xi,yi) with control register. Finally, classification can be done by interaction via interference (|S3 >) between Te(r) and Tt(r) as shown in Eq. (7) in which fi(x’) is used to store the estimate of the target variable which depends on the ‘ith’ sub-sample and test set x’ and expectation measurement (<M>) on target qubit is determined using Eq.  (8) where several weak learners (B) is equal to 2^n^ which provides ensemble prediction since the final result is obtained from the average of predictions of all weak learners. If classes of the target variable are encoded into two basic states then it is possible to determine the final prediction value by using a single qubit measurement as shown in Eq. (9) in which x_0_ and x_1_ are the average probabilities for the test set x’ to be classified in class ‘0’ and class ‘1’ respectively.

5 $$|{\text{S}}_{{1}} > \, = \, ({\text{h}}^{{\text{n}}} \otimes {\text{u}}\left( {{\text{x}},{\text{y}}} \right) \otimes {1} \otimes {\text{u}}\left( {{\text{x}}^\prime } \right) \otimes {1})|{\text{S}}_{0} >$$

6 $$|{\text{S}}2> =\left(\frac{1}{\sqrt{{2}^{n}}}\sum_{i=1}^{{2}^{n}}|i>|x,y>|xi,yi>\right)\otimes |{\text{x}}^{\prime}\hspace{0.17em}>\hspace{0.17em}\otimes |0\hspace{0.17em}>$$

7 $$|{\text{S}}3> = \left(\frac{1}{\sqrt{{2}^{n}}}\sum_{i=1}^{{2}^{n}}|i>|x,y>|xi,yi>|{x}^{\prime}>|fi({x}^{\prime})\right)$$

8 $$<{\text{M}}> = \frac{1}{B}\sum_{b=1}^{B}fb=fb({x}^{\prime}|\left(x,y\right))$$

9 $${\text{fb}}=\sqrt{{x}_{0}}|0> + \sqrt{{x}_{1}}|1>$$

## Results and discussions

In this section, the results of quantum approaches performed over quantum simulators are presented. The QANN has been implemented using the PennyLane python library which has inbuilt methods for optimizing rotational parameters of quantum gates. The experiment is performed over the comprehensive data set described in Table 2 which contains 1190 instances among which 629 instances are labeled as HD^+^ (Heart Disease Positive) and 561 instances are labeled as HD^−^ (Heart Disease Negative). Likewise, traditional ANN has been implemented with the same data set using Keras API. In traditional ANN, it contains input layers with 8 nodes, two hidden layers with 5 and 3 nodes respectively, and the output layer has one node since each input is associated with a class label of binary value. Thus, the total number of parameters in traditional ANN to be optimized is 58 and the class label is predicted by using the sigmoid activation function. Both QANN and ANN are implemented with a set of hyperparameters as shown in Table 5.

**Table 5:** Hyperparameters.

S.no	Parameters	Values
1	Optimizer	Adam optimizer
2	Learning rate	0.01
3	Momentum	0.9
4	Kernel initialization	Standard normal distribution
5	epochs	100
6	ḉ	1
7	ṕ	l2 regularization
8	ṫ	0.0001
9	Qubits	3

Similarly, traditional SVM and Bagging Ensemble are implemented using Scikit-learn python library with the following tuned hyper-parameters of SVM as shown in Table 5 in which ḉ denotes the regularization parameter, ṕ represents penalty type, and ṫ denotes the tolerance parameter. ḉ defines the misclassification rate and the regularization parameter controls the strength of regularization which is inversely proportional to ḉ and ṫ defines the termination criterion whereas quantum SVM and QBE are implemented using Qiskit Library with the number of qubits = 3. Moreover, an overall experiment is carried out with Python and Keras API of Tensorflow 2 on a Ryzen7-3.20 GHz Processor, Graphical Processing Unit (GPU) NVIDIA RTX 3060 with 6 GB and 16 GB RAM respectively. The performance of the QuEML framework has been evaluated^45–47^ in terms of precision (P), accuracy (A), recall (R), sensitivity (Se), specificity (Sp), F1score (F), and training time (T) and results are populated using fivefold cross validation technique. Further, the given data set has been split into 60%, 20%, and 20% for training, validation, and testing respectively. Mathematically, these performance metrics are computed using Eqs. (10), (11), (12), (13) and (14) where N(T +) denotes the number of True Positives, N(T-) denotes the number of True Negatives, N(F +) denotes the number of False Positives and N(F-) denotes the number of False Negatives.

10 $${\text{P}}=\frac{N(T+)}{N\left(T+\right)+N(F+)}$$

11 $${\text{A}}=\frac{N\left(T+\right)+N(T-)}{N\left(T+\right)+N\left(T-\right)+N\left(F+\right)+N(F-)}$$

12 $$\mathrm{R \,or \,Se}=\frac{N(T+)}{N\left(T+\right)+N(F-)}$$

13 $${\text{Sp}}=\frac{N(T-)}{N\left(T-\right)+N(F+)}$$

14 $${\text{F}}=\frac{2*P*R}{P+R}$$

The confusion matrix is shown in Tables 6 and 7 which shows the classification ability of Quantum Enhanced Machine Learning approaches (QuEML) and Traditional Machine Learning approaches (TML). From the above tables, it has been observed that QBE is predicting 1144 samples correctly from the given samples of 1190. It is 0.42% and 1% higher than the QSVM and QANN respectively. When comparing the classification performance of QuEML with TML, TML approaches are performed with an average misclassification rate of 2.96% that is 211 samples are misclassified which is around 0.29% higher than QuEML techniques.

**Table 6:** Confusion matrix of QuEML.

QuEML (in %)	N(T+)	N(T-)	N(F+)	N(F−)
QBE	50.50	45.63	1.513	2.35
QSVM	50.08	44.87	2.269	2.77
QANN	49.50	43.45	3.697	3.36

**Table 7:** Confusion matrix of TML.

HTML (in %)	N(T+)	N(T−)	N(F+)	N(F−)
BE	50.17	45.13	2.02	2.69
SVM	50.00	44.96	2.18	2.86
ANN	49.16	42.86	4.29	3.70

Table 8 shows how QuEML outperforms all baselines in terms of Accuracy (A), Precision (P), Recall (R), Specificity (Sp), and F1 Score (F) where A measures the classification ability and QBE shows excellent prediction performance with 0.953 of accuracy because of its ensemble nature which is around 1.1% and 3.2% greater than QSVM and QANN respectively. The term P measures the false positive rate of QuEML where QBE, QSVM, and QANN have the precision rate of 0.971, 0.957, and 0.930 respectively. R shows the prediction ability of quantum techniques in terms of true positive rate. Totally, 629 positive samples are used for experimental purposes in which the proposed quantum approaches predicted around 595 positive samples as positive. Similarly, the proposed quantum frameworks have predicted an average of 531 samples as negative from the 561 negative samples. As per the assertion, the higher rate of precision and recall automatically increases the F1 score. So, QuEML has an F1 score of 0.950 which is closer to 1 indicating excellent classification ability of quantum approaches. Table 9 shows the overall performance of TML in which it has been observed that the overall accuracy of TML is an average of 0.941 which is around 0.6% lesser than QuEML. Likewise, the P, R, Sp, and F rate of TML is around 0.7%, 0.4%, 0.7%, and 0.6% lesser than QuEML. Overall, QuEML is attaining a little bit greater performance (0.6% greater) than TML because of its quantum nature.

**Table 8:** Overall performance of QuEML.

QuEML	A	P	R	Sp	F
QBE	0.961	0.971	0.955	0.968	0.963
QSVM	0.950	0.957	0.948	0.952	0.952
QANN	0.929	0.930	0.936	0.922	0.933

**Table 9:** Overall performance of TML.

TML	A	P	R	Sp	F
BE	0.953	0.961	0.949	0.957	0.955
SVM	0.950	0.958	0.946	0.954	0.952
ANN	0.920	0.920	0.930	0.909	0.925

**Figure 7:**
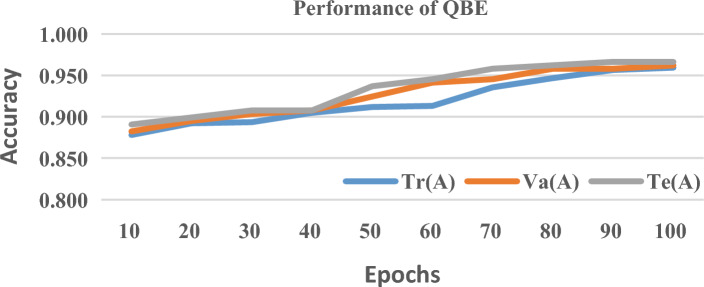
Tr(A) vs. Va(A) vs. Te(A) of QBE.

**Figure 8:**
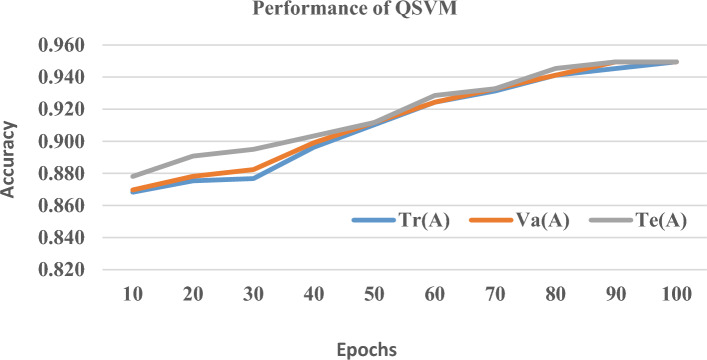
Tr(A) vs. Va(A) vs. Te(A) of QSVM.

**Figure 9:**
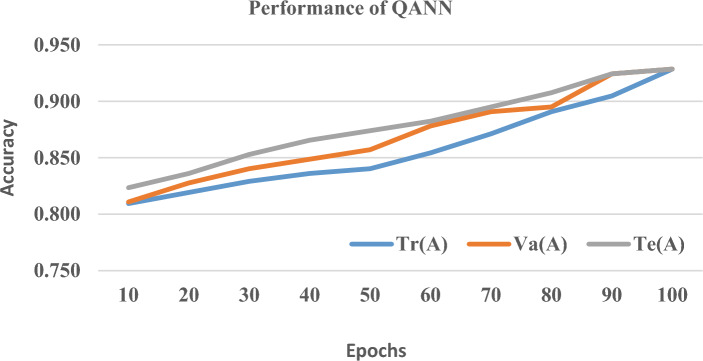
Tr(A) vs. Va(A) vs. Te(A) of QANN.

To measure the generalization performance of the proposed quantum framework, the given data set is split and training Tr(A), testing Te(A) and validation V(A) accuracy is measured at each epoch from 1 to 100 which is shown in Figs. 7, 8 and 9 At the 100th epoch, QBE has Tr(A), V(A), and Te (A) rates of 0.959, 0.962, and 0.966 respectively. Likewise, QSVM and QANN have increased the accuracy rate from 0.872 and 0.815 to 0.950 and 0.929 respectively. From the above charts, it is visible that the proposed quantum framework is free from overfitting which shows its excellence in generalization performance since it greatly reduces the distance between Tr(A), V(A), and Te(A).

Moreover, the major reason to implement the QuEML is to speed up the classification performance. In this paper, two methods were enforced to reduce the time taken to train the model such as CSE-based feature selection and quantum-enhanced machine learning approaches. The results populated in Table 10 show the training time Tr(t) of QuEML which is compared with Tr(t) of TML in microseconds. It is demonstrated that the proposed QuEML uplifted the performance in terms of Tr(t) which is around 1.3 times greater than the traditional machine learning approaches. From Table 9, SVM has taken less time 755.5 µs for training than ANN and BE. But when employing quantum principles in TML, QANN has achieved a remarkable performance of 616.6 µs in training time than QBE and QSVM. In the proposed framework, the CSE method is used to eliminate the irrelevant features from the given data set which shortens the training time considerably as shown in Table 11. After applying CSE, QuEML is fed with an Optimized Feature Set (OFS) which has reduced features. Such a reduced feature set improved the performance of QuEML in terms of training time. As shown in Table 10, QBE with OFS has consumed 713.4 µs which is around 47.6 µs faster than QBE with FFS (Full Feature Set). Similarly, QSVM and QANN with OFS trained faster (679.9 µs and 616.6 µs) than QSVM and QANN with FFS (690.7 µs and 666.4 µs).

**Table 10:** Training time in µs.

QuEML	Tr(t)	TML	Tr(t)
QBE	713.4	BE	951.2
QSVM	679.9	SVM	755.5
QANN	616.6	ANN	880.8

**Table 11:** Performance of CSE in terms of Tr(t) in µs.

QuEML	Tr(t)_OFS	Tr(t)_FFS
QBE	713.4	761.0
QSVM	679.9	690.7
QANN	616.6	666.4

## Conclusion

The accurate diagnosis of heart disease is critical in reducing mortality rates. This paper reviews various computer-aided diagnosis (CAD) systems developed using machine learning (ML) approaches, highlighting their strengths and weaknesses. From the study, it has been found that traditional machine-learning approaches are further enhanced by employing quantum principles. To prove this, an experimental study has been carried out over the Kaggle heart disease dataset. The performance evaluation is made against QuEML vs. TML in which the traditional bagging ensemble (BE) has performed well with an accuracy of 95.3% which is 0.3% and 3.3% greater than traditional SVM and ANN. But in the case of training time, SVM performed better with a training time of 755.5 µs which is around 160.5 µs than traditional ANN and BE approaches. Overall, QuEML approaches yielded average accuracy of 0.947 which is around 0.6% greater than TML, and also QuEML has attained a remarkable performance in training time which is around 192.5 µs greater than TML. From the experimental study, it is proved that quantum-enhanced machine-learning approaches outperform traditional machine-learning approaches in terms of accuracy and computational time.

Even though the QuEML is beneficial for accuracy and speed up the diagnosis process, still it poses several practical challenges. The qubits may drop their quantum properties such as entanglement which leads to loss in stored data since heat and light causes quantum de-coherence. Similarly, incorrect qubit rotations lead to an inaccurate result which causes misdiagnosis. In the future, the performance of the heart disease diagnosis system can be boosted further by integrating deep learning approaches with QuEML.

## Data Availability

The datasets analysed during the current study are available in the kaggle repository, https://www.kaggle.com/datasets/sid321axn/heart-statlog-cleveland-hungary-final.
